# Emerging Complexity in Distributed Intelligent Systems

**DOI:** 10.3390/e22121437

**Published:** 2020-12-19

**Authors:** Valentina Guleva, Egor Shikov, Klavdiya Bochenina, Sergey Kovalchuk, Alexander Alodjants, Alexander Boukhanovsky

**Affiliations:** National Center for Cognitive Research, ITMO University, 197101 Saint Petersburg, Russia; guleva@itmo.ru (V.G.); egorshikov@itmo.ru (E.S.); kbochenina@itmo.ru (K.B.); alexander_ap@list.ru (A.A.); boukhanovsky@mail.ifmo.ru (A.B.)

**Keywords:** distributed intelligent systems, complex systems, natural intelligence agent, artificial intelligence agents, multiagent systems, self-organization, reinforcement learning, quantum intelligence

## Abstract

Distributed intelligent systems (DIS) appear where natural intelligence agents (humans) and artificial intelligence agents (algorithms) interact, exchanging data and decisions and learning how to evolve toward a better quality of solutions. The networked dynamics of distributed natural and artificial intelligence agents leads to emerging complexity different from the ones observed before. In this study, we review and systematize different approaches in the distributed intelligence field, including the quantum domain. A definition and mathematical model of DIS (as a new class of systems) and its components, including a general model of DIS dynamics, are introduced. In particular, the suggested new model of DIS contains both natural (humans) and artificial (computer programs, chatbots, etc.) intelligence agents, which take into account their interactions and communications. We present the case study of domain-oriented DIS based on different agents’ classes and show that DIS dynamics shows complexity effects observed in other well-studied complex systems. We examine our model by means of the platform of personal self-adaptive educational assistants (avatars), especially designed in our University. Avatars interact with each other and with their owners. Our experiment allows finding an answer to the vital question: How quickly will DIS adapt to owners’ preferences so that they are satisfied? We introduce and examine in detail learning time as a function of network topology. We have shown that DIS has an intrinsic source of complexity that needs to be addressed while developing predictable and trustworthy systems of natural and artificial intelligence agents. Remarkably, our research and findings promoted the improvement of the educational process at our university in the presence of COVID-19 pandemic conditions.

## 1. Introduction

The concept of distributed intelligence (DI) is currently known for decades [1]. Within the multitude of works and directions of DI, the distribution is applied to knowledge, information, action, and resources [2]. The DI solutions in many problem domains are growing in scale, achieving the support of big data volumes, ubiquitous computing resources, etc. This opens new opportunities and new behavior of DI. Initially, the DI area’s main focus was distributed decision-making and stakeholders’ (humans) interaction. Still, the advances of artificial intelligence (AI) also influenced the area by introduction of the idea of distributed artificial intelligence (DAI) [3,4], discussion of cognitive abilities in distributed agents [4,5], and incorporating cyber–physical DI, sensors, intelligent agent, Internet of things (IoT), etc. Considering artificial intelligence (AI) agents within the DAI approach, an emergent collaboration and self-organized interaction often appear, pushing a swarm intelligence [6] and related ideas to an entirely new level. Emerging collaboration in bots is observed and even hired in different areas (Wikipedia editing [7], e-Government [8], etc.). Together with a deeper understanding of human–bot interaction [9,10], it leads to the idea that systems with AI agents and humans (expert, users, operators, etc.) will enormously grow in scale and obtain the features of complex systems [11] in the near future. In addition to the emergent structure, such phenomena as high-order interactions [12] and network information percolation [13] may appear in such systems. 

On the other hand, systems of AI agents and human operators represent a novel class of information systems that can be considered from two points of view. First, the functional characteristics of such systems are based on the effectiveness of internal information exchange. One can consider a DI system as a distributed self-organizing information system, which is described through information theory approaches [14,15]. Second, an information system as an object for design and development rely on exploitative quality measures and indicators [16,17]. Still, with a level of complexity achieved in large distributed intelligent systems (DIS), the performance measures strongly depend on emergent characteristics and agent behavior. 

Complex systems meet AI systems in recent studies [18]. The authors apply machine learning techniques to problems met in complex networks and nonlinear dynamical systems, such as resilience analysis, control and optimization problems, evolution forecasting, etc. Still, the questions arise: Can intelligent systems demonstrate the complexity? And how do complex dynamics appear from local dynamics of natural and AI agents?

Finally, it is worth noticing DIS applicability in the quantum domain. This domain is an area where the quantum approach to human cognition [19,20] and AI [21] meet new facilities served by current quantum communication [22] and quantum computing technologies [23]. Full-scale quantum internet [24] and quantum IoT [25] may be one of the results for the quantum approach to the DAI problem. Undoubtedly, the complexity of quantum (or quantum-like) DAI systems represents an entirely new task established at the border of both classical and quantum information technologies, AI, and cognitive sciences.

Within the presented research, we systematically review both existing (reported in the literature) and perspective issues coming from DIS complexity and emergent phenomena from the perspective of internal functional structure and external performance characteristics. We propose an approach to describe the dynamics of DIS and explore the proposed approach using motivational models. Finally, we discuss the implications of effects observed in DIS for the development of reliable solutions and compare DIS with other classes of systems demonstrating complexity effects.

The paper is arranged as follows. In Section 2, we shortly establish the current state of the art for DIS. We are briefly reviewing the key features of DIS and represent their importance for advanced intelligent systems development. In particular, we discuss the relevance of DIS to multiagent systems (MAS), to distributed expert systems and, to cognitive architectures available now. We consider an agent-based machine learning approach to elucidate DIS that possesses several networked intelligence agents. The quantum approach to DIS is also discussed. In Section 3, we establish our model of DIS as a combined system of natural and AI agents that interact with each other. In particular, we give a clear mathematical description of DIS and establish intelligence agents’ characteristics and rules for their interaction and communication within a network environment. We examine our model experimentally in the framework of the Avatar project implemented at ITMO University recently. This project aims to help the students reduce some routine work in the framework of online regime education, which represents a necessary and important educational tool for the current pandemic of COVID-19. In Section 5, we substantially discuss the benefits of our experiment outputs considering complexity problem for the DIS. In conclusion, we briefly summarize the results obtained.

## 2. State of the Art

An intelligent system (IS) is mainly aimed at solving human-level problems with high uncertainty, vague definition, lack of data, and other imitations. IS’s key common features are reasoning using several common components including knowledge base, database, inference engine, knowledge acquisition procedures, models for model-based reasoning, etc. An IS may also include experts (as knowledge sources), operators, and other human agents (including target user or decision-maker). Starting from this, we consider DIS as a more comprehensive class of IS, providing intelligent *personal* solutions for many users and containing *interacting parts* within the system. DIS aim at optimizing user satisfaction at minimal costs. In this way, they can contain components of DIS-like algorithmic and knowledge bases and decision-making techniques. Nevertheless, data structure and volumes in the context of personal orientation and privacy restrictions raise the necessity of system distribution, which can also increase learning speed, functional quality, and resource efficiency. In addition, the structure of interaction between intelligent agents, representing DIS, in combination with local dynamics, may result in emergent phenomena and critical effects, influencing DIS functional properties. 

To explore how DIS meet complexity in terms of emergent phenomena brought by the structure of interactions, we performed a search for complexity in IS, DI, and network effects. Special attention was given to the possible emergent phenomena such that they focused on interactions with a user, or structural effects, or distributive effects, or intelligence levels.

Below, we consider various systems in the framework of the DI technology paradigm (ref. [2]) and analyze some of their properties that exhibit novel and still unexplored complexity features of AI due to its distribution in various socially oriented networks. In this way, our review results in the following sections: concerned sensor networks (physical distribution of resources), multiagent systems (action distribution), cognitive architectures and expert systems (knowledge and intelligence distribution), quantum intelligence (information distribution), and multiagent machine learning (model distribution), comprising intelligent agents and network effects. We try to disclose different kinds of complexity and criticality in DIS-related areas.

### 2.1. Sensor Network

Sensor networks are the simple class of DIS. They are multiagent, distributed, networked; they may contain intelligent agents as sensors and are applied for environmental monitoring. They contain a communication level with corresponding communication protocols from a dynamics viewpoint, contributing to emergent effects [26]. Nevertheless, the main problems of sensor network configurations are the prolongation of network lifetime and costs reduction, which both arise at different layers, from physical and network to application, and require nodes’ self-organization abilities. Therefore, the complexity here is computational complexity and resource consuming.

A wide variety of applications requires an architecture design of sensor networks [27]. Functionality and process dynamics on sensor networks are often related to their topological structure, affecting the security of information flows [28]. In particular, node capture attack blocks single nodes in a network, reducing communication paths and connecting network resilience to such attacks with dynamics on networks and topological properties [29]. Wu et al. [30] suggest a graph partition model for decreasing computational complexity in fault diagnosis tasks related to transmission on networks.

Nazemi et al. [31] suggest a framework for flow optimization in sensor networks. They consider it as a nonlinear optimization problem and then suggest a decomposition of a transmission tree into a linear graph, which significantly decreases problem complexity. Cyclomatic complexity [32] is considered as a technique of time-consumption estimation of security algorithms on sensor networks. The authors emphasize the importance of the efficiency issues in sensor networks due to their heterogeneity and complexity. An efficient intrusion detection method is also considered by Khanna et al. [33], who suggest how to choose single sensors as monitoring nodes. Node fitness is evaluated dynamically by genetic algorithms and considers the combination of node placement (topological features) and their attributes.

The sensor networks environment has tangible resource restrictions. In this way, several functionality-related tasks, security, and fault tolerance assessments are mostly related to resource-consuming problems.

### 2.2. Multiagent Systems

Multiagent systems (MAS) include the representation of individual agents, their interactions, and the environment [34], and in this way, they reflect the distributed organization, which is peculiar to DIS. The agents’ appropriate definition depends on what they represent, for example, people, machines, particles, organisms, or computing systems. The agent’s capabilities include communicating with other agents, a mechanism for perceiving the environment to fix the state of the properties of interest, and a mechanism of exposure or influence on others and the environment. The knowledge model collects the necessary information for the agent to work, including its state and the state of the environment and other agents. For example, knowledge models have been proposed using a fuzzy cognitive model in [35] or ontologies in [36]. Research on intentional systems [37] has led to more sophisticated models that simulate how people make decisions. In the Belief–Desire–Intention (BDI) model, the information perceived by the agent as facts represents beliefs, and desires and intentions represent the totality of future states that the agent can achieve, with the difference that the agent is only committed to working toward intentions [38]. Interaction models control the collective behavior of the system and provide communication between agents. In addition, such models make it possible to manage the interdependencies between the actions of agents [39].

The MAS area is closely related to DIS, and it started as an approach to launch complex distributed problems by dividing operations between cooperative computing units (agents) who plan, reason, and communicate to complete their part of the solution. The essence of such systems lies in their ability to provide solutions beyond each participating agent’s capabilities. The mechanisms of coordination and cooperation of agents as a society are fundamental characteristics of any MAS [40]. For example, the authors of [41] proposed a long-term adaptive distributed intelligent systems model that combines an organization theory and multiagent paradigm—ViaBots. Each part of the simulated system within ViaBots is designed as an autonomous agent. In turn, the entire model is considered as a MAS. The simulated model allowed the multi-robot system to adapt to the number and characteristics of the robots available and changes in the parts processed by the system.

MAS tends to reflect the system under study in terms of internal communication agents and the surrounding physical or informational environment. MAS can implement some features of DIS, such as the collective behavior of many agents without centralized coordination (with catching the emergent phenomena), high-order behavior of groups, distributed exploration of the environment, etc. Still, MAS rarely considers explicit collaboration between agents and agents’ dynamic learning during their experience within a MAS. In addition, they are not able to fully reflect the interaction of natural and artificial intelligence agents.

### 2.3. Distributed Expert Systems and Cognitive Architectures

Here, we consider studies, focused on intelligence, cognitive ability, and adaptation to new tasks. In this way, possible critical effects are related to computational and physical level, and networked complexity is related to architectures of cognitive systems, aimed at the organization of effective information spread between heterogeneous components. Distributed expert systems are combinations of entities of different nature aimed at intelligent problem solutions. In this way, single components may differ in their function, and the network of interactions is not necessary. Related studies explore architectural issues and fault tolerance questions.

One of the approaches where distributed expert systems mimic human professional activity behavior is a formal (mainly symbolic) expression of reasoning and knowledge in expert systems [42]. Having a long history of success in particular applications, the typical approach of expert systems faces known limits in the generalization of the reasoning process and influence of uncertainty in problem definition. Thus, to be more general, a “single-expert” system should consider the cognitive abilities of a human being [43]. This idea stimulates cognitive architectures’ development as a basic framework for intelligent agent reasoning [44].

The idea of combining intelligent agents with different architectures to the system, which provides adaptability to environments and problems, is introduced in [45]. They decompose agent architecture to adapt its component to tasks. In this way, each problem is decomposed into subtasks and characterized by problem spaces, goals, states, and operators. The decision-making procedures were introduced using propositional logic [46], determining the chosen solution optimality.

A review of cognitive architectures from the point of cognition theories, psychology, and artificial intelligence as an application is performed in [44]. The authors emphasize cognitive architectures’ necessity to adapt to environmental changes and use knowledge efficiently to perform new tasks and present a taxonomy of cognitive architectures considering their representation and processing method. They also distinguish cognition, learning, memory, perception, and reasoning components in these types of architectures. One of the cognitive platforms containing multiple agents is the SOSIEL (Self-Organizing Social & Inductive Evolutionary Learning) Platform [47]. The proposed cognitive architecture comprises decision-making, cognitive, and memory blocks, being in correspondence with cognitive theories at all stages. The connection of humans, machines, and the environment [48] is also reviewed in a vision article, showing the authors’ view on intelligent systems development in 5–10 years.

In contrast to distributed expert systems architectures’ functional design, fault tolerance is also considered an essential requirement, which is also related to distribution. In this way, *the criticality* of IS is considered as their resilience to faults. Faults specification is provided in [49] with a division to hardware, environmental, and cognitive faults in systems and possible detection methods. A set of definitions, including adaptability, sustainability, complexity, etc., is described in the context of a framework, aimed at developing digital ecosystems [50]. Understanding systemic complexity is concerned, suggesting a model checking framework for architecture development and understanding during complex, intelligent system modeling [51].

In this way, this kind of system implements intelligence, contains heterogeneous components providing functional properties, and meets complexity, adaptability, and resilience as functional necessities, mostly at the physical level.

### 2.4. Multiagent Machine Learning

This section reviews DIS from the point of distributed algorithms. In this way, we observe ensemble learning and multiagent learning. On one hand, multiagent learning may consider network effects on learning process, but on other, it can consider interactions with natural intelligent agents (NIA) via a reinforcement learning process (multiagent reinforcement learning). Agents are learned to satisfy user needs and are served by machine learning techniques. In this way, the system is homogeneous, intelligence is decentralized, and self-organization may exist. The structure of agent interactions may affect learning quality as a ground for knowledge spreading through the system, which is also explored by some authors.

*Ensemble-based approaches* could be considered as a general reasoning, prediction, or forecasting of semantically identical objects (agents, models, classifiers, and datasets). One of the widespread implementation classes within this approach is ensemble learning, which implements an interaction between learning algorithms concerning multiple models and datasets to forecast an outcome by aggregating several predictions. Aggregation can be performed as a combination of predictor results for regression [52] or selecting a sole outcome for classification [53].

Today, ensemble-based solutions are implemented in various aspects and areas [54,55,56]. Ensemble modeling methods have been long and widely used in the simulation. For example, a hydro-meteorological prediction could be considered as one of the most adopted areas for ensemble-based approaches. Ensemble modeling is used to manage uncertainty in complex systems [52] or to utilize data from different sources [57]. Ensemble learning usually shows a higher performance than a single model predicting general outcomes [58,59]. In [60], a neural network ensemble model trained by negative correlation was used as a “model builder agent” in a four-layer multiagent system architecture to predict free swelling index. A cooperative training algorithm showed the potential to improve the generalization ability. An evolutionary approach was used in [61] to build an ensemble structure, i.e., to compose optimal sets of models and input variables. Evolutionary-controlled ensembles provide better performance than a single model learning in scoring predictions. Therefore, ensemble learning can improve prediction quality using the synergy of its elements.

Effects of *network topology on learning dynamics* is studied by Hao et al. [62], who explore individual and joint learning and four kinds of interaction topologies in the reinforcement social learning tasks. The authors measure the learning performance for each strategy and topology in a two-player cooperative Markov game and show that an individual learning strategy makes learning slower, while the limit performance is approximately the same. Random network topology demonstrates a twice as low performance; ring and small-world structures increase learning speeds. Lattices and scale-free networks were explored in strategy switching and cooperation effects on decision-making in multiagent systems [63]. The authors use evolutionary game theory as an instrument reflecting conflicts of agent interests. The number of agents having each kind of strategy is evaluated with differential compartmental models and network topologies. The experiments show no effect on the observed dynamics, efficiency significantly, and strategies consumption patterns.

In contrast to strategy switching, knowledge sharing issues, performed in a logic form with propositional logic inference, fulfilling a knowledge base, are sensitive to network density, since it affects all agents’ access to the knowledge system [64]. Experiments confirm this with SMILE [65,66] protocol for the consistency check of hypotheses (with initial examples) and groups of agents. 

*Multiagent reinforcement learning* (MARL) exploits multiple agents and interactions between agents of natural and artificial intelligence. Nevertheless, their complexity is often restricted by a single natural intelligence agent (NIA), served by the whole system, despite possible effects of network topology connecting artificial intelligence agents (AIAs). MARL focuses on finding optimal strategies for agents to operate in an environment with other learning agents. An important feature is the settings it solves [67]. In this way, MARL algorithms can be divided into three groups: fully cooperative (agents collaborate to optimize overall long-term returns), fully competitive (the return of agents sums up to zero), and combined (both cooperative and competitive agents). 

Another essential feature of MARL is the topology of connections between agents. Gupta et al. [68] demonstrate a connection of training results to the underlying network topology. Sheng et al. [69] argue that the hierarchical network structure is more efficient for multiagent learning than stars, full graphs, and block models. Hu et al. [70] use general sum stochastic games to model interactions between agents and Markov decision processes as a reward method. Some studies enhance the results using agent cooperation but consider random structures of interaction, as in this study about intelligent traffic light control using distributed multiagent Q-learning [71].

The problem of communication strategies and languages learning in multiagent systems, aimed at optimizing summarized utility function, but without consideration of structural network pattern is the studies in Cao et al. [72], Mordatch and Abbeel [73], Havrylov and Titov [74], Gupta, and Dukkipati [75]. The concurrent learning of multiple agents opens a problem of environmental non-stationarity, which is solved using modern multiagent learning algorithms, a compilation of which can be found in [76]. Finally, the global dynamics of reasoning in intelligent agents’ populations can be formalized with game–theoretical local dynamics and the Markov decision process. Bloembergen [77] observes possible scenarios of such evolution from the point of populations comparts taking decisions.

In this way, one can see networked intelligent agents, affecting systemic properties, with potential critical effects, and feedback dynamics from users, corresponding to each intelligent agent. Nevertheless, the potential “criticality” is restricted by several factors: by effects of network topology on learning speed and quality (and this is shown by empirical results); and by compartmental learning dynamics without networking effects. DIS-related studies are expected to explore dynamics driven by local dynamics between users and intelligent agents as well as the potential effects of network interaction.

### 2.5. Quantum Approach to DIS

Quantum science serves as a new methodological approach to advanced DIS’s discussed in this work. In the quantum domain, the DIS problem may appear in different ways. First, it occurs at the modeling level resolving natural (humans) intelligence peculiarities. We can provide the modeling of NIAs (humans) by means of quantum probability theory and non-boolean logic that may be a useful tool for the solution of some specific decision-making problems in practice under risk and uncertainty [78,79]. In particular, quantum probabilistic approaches may be useful for resolving and the explanation of judgments and decisions associated with so-called “cognitive biases”, which appear as a result of a violation of the sure-thing principle [80]. The “interaction” of humans with the classical environment represents a new, quantum-like paradigm in modern social and cognitive sciences [81]. The classical environment, which may be represented by some AIAs, recommendation systems, information retrieval systems, etc., specifies some quantum-like (contextual) paradigm that may also be resolved in the framework of quantum instruments approach [82]. Second, the quantum approach may be applied for the description and characterization of AIAs. In particular, the so-called quantum machine learning approach aims to study agent-based machine learning tasks [83]. Due to uncertainty that is inherent to the quantum approach of description ab initio, agents are always situated and interacting within some environment [84]. Here, we would like to underline two basic layers for the quantum agent-based machine-learning paradigm. First, the agent is guided by a quantum algorithm or, simply, established by a quantum computer that helps to solve relevant classical (machine learning) tasks. In this case, we can expect to obtain speedup for the solution of some classical computational problems due to quantum algorithms implementation. Second, we can suppose that the agent is guided by classical rules and relevant algorithms aimed at resolving quantum problems that are characterized by some “environment”. In this case, active learning agents help, as an example, to recognize some new unusual features of quantum devices, schemes, algorithms, etc. [85]. It is evident that current social, technological, and other networks provide new reality and digital ecosystems for quantum (or quantum-like) DIS, which are not studied systematically yet. In particular, current quantum communication technologies serve new platforms and algorithms for information transmission in complex networks [86]. In this case, the quantum speedup problem for information transport is not so obvious and depends on the topological characteristics and complexity of used networks [87]. On the other hand, under the limited time condition, in the presence of uncertain (or overloaded!) vague information, humans as decision-making agents of the WWW (World Wide Web) and the Internet become subject to external informational influence and manipulation, which cause some irrational decisions. Studies of these processes in social systems, which exhibit new complexity and possess phase transitions, are just at the beginning now [88]. Recently, it is shown that quantum phase transitions that occur in the zero-temperature limit may represent some manifestation of quantum-like behavior of collective emotions in the network community [89]. Although the investigation of quantum effects in complex networks is not a goal of this work, the study of contextually dependent phenomena, which is carried out in special DIS systems exhibiting some phase transition features, can be very useful in the framework of the quantum approach for further studies.

### 2.6. Valuation and Comparison

The observed research areas demonstrate explicit components, which are also met in DIS, and these are related to significant functional properties. In this way, the related studies are focused on the influence of these components on the observed dynamics. MAS explore agent parameters; networked systems are focused on topological effects. Some systems combine factors, such as networking and intelligence, and explore their co-evolving impact. MARL meets intelligence and topological effects; nevertheless, centralized structures initiated by a knowledge-based connection diminish network effects. In addition, the introduction of multiple agents in MARL approaches often aims at computational complexity reduction. 

Table 1 compares various systems met by the properties of DIS. The table explains how they concern the considered question from different sides, between MAS, complex networks, and expert systems. In addition to the areas considered earlier, we include complex networks as well-developed [90] and reviewed in the scope of complexity (see for example [91,92]). We also explicitly display ensemble learning considered in Section 2.4 as a form of model-based distribution due to its significant difference and reduction in the number of observed features but sustainable and widely used in practice.

DIS combine components met and provide intelligent properties brought by single distributed agents that are networked by communication paths. In addition, they must have feedback connections with agents of natural intelligence, which is a necessary component for individual tuning in contemporary AI tasks. Evolutionary dynamics of interactions of natural and artificial intelligence agents are explored only in the context of reinforcement learning [77] and seems to bring additional complexity to networks of agents as if we had multiple environments. 

We can identify several drivers that force moving from one horizon to the next one. Among them: the growth of a number of intelligent components of different classes within existing and forthcoming systems; the appearance of multiple data being stored, transferred, and processed within the systems; the ubiquitous presence of information technologies components (computers, sensors, robots, etc.); multiple stakeholders and active agents in online systems, and many others.

Table 1, which compares DIS properties, is also valid for referred systems in the quantum domain. However, in this case, we should take into account additional facilities provided by quantum information transmission and communication [93]. In particular, quantum entanglement that may occur for network nodes, AIAs, and information carriers (photons) introduce kindly new features in complexity problem. For example, we can refer here to quantum dense coding, quantum repeaters, and quantum channels (such as teleportation) capabilities, which may be explored and built in complex networks [94]. At present, due to various technological limitations, the use of quantum communication channels in large-scale complex networks is minimal; see Table 1. Quantum communication networks, which are currently starting to implement classical AIA facilities, generally are realized at the principle of proofs level [95]. Their complexity and capabilities are not yet known in detail [96].

The above-mentioned drivers introduce complexity to systems by bringing additional feedback links. Understanding of their interconnections and contributions to systemic dynamics allows making judgments about systemic properties at the design stage before implementation. Therefore, we formalize the contents of DIS and its dynamics and demonstrate how emergent phenomena are influenced by single elements and their interactions, which is disclosed further in the article.

## 3. DIS: Generalized Motivational Model

As it was mentioned in the previous section, there is a multitude of approaches to describe and implement DIS in various problem domains. Moreover, within the evolved areas of complex distributed systems modeling and simulation, we identified several drivers forcing the development of more complicated systems, which could be described as DIS. In this section, we introduce general formalization, which is aimed to describe various forms of DIS as a generalization of IS within the scope of the growing complexity of systems with diverse classes of agents in it.

### 3.1. Definitions and Features

A distributed intelligent system (DIS) is built as a decentralized system of interconnected agents, each of which may be considered as an autonomous IS. Agents may be of a single role or have different roles. External connection for agents in such a system may represent either information exchange with other agents in the system or connection to the “outer world”. At the same time, each of the agents could be IS focused mainly on one component/aspect of implementation, e.g., expert assisting modules with a key feature of knowledge acquisition may be an autonomous agent within the DIS.

**Definition** **1.** *A distributed intelligent system is considered as a tuple* DIS=〈A,G,K, E〉 *, where* A *is a set of agents within a DIS;* G *is an interconnection graph that defines information exchange within a DIS, K is a system-level knowledge base (including general inference engines, self-organizing procedures, etc.), and* $E\subset {\mathbb{R}}^{n}$ *is event environment where DIS is operating.*

Within our study, we consider DIS, which shows the features and behavior of complex systems [11,70]. Key research questions arise from the interconnection of intelligent characteristics of agents in DIS and the complex nature of a system. The following key characteristics of DIS may be defined:

1. Commonly, a DIS consists of agents A of various roles. This includes agents with an explicit and essential role of human interaction (furtherly called natural intelligence agents, NIA) and agents with the primary role of AI components (furtherly called artificial intelligence agents, AIA). In the general case, $A=\left\{a_{i}\right\}\cup \left\{u_{i}\right\}$, where $\left\{a_{i}\right\}$—a set of AIAs (Definition 2), $\left\{u_{i}\right\}$—a set of NIAs (Definitions 3 and 4).
2. Decentralized network-based nature of DIS. In a large-scale DIS, one can consider its structure G as a complex network (Definition 5) where the emergent structures and topology influence both information exchange procedures and high-level performance characteristics of DIS. In addition, in a fully decentralized system, usually K=∅, so the agents behave under self-motivation and self-regulation.
3. Agents and subsystems within a DIS may have different goals, objective functions, behavior models, etc. (see Definition 5). It is more natural for NIA; still, AIA optimizing their outcomes may select different behavior strategies too as a reaction to the environment E, and according to the structure of G and other agents’ behavior.
4. The self-organization of large-scale DIS enables the adaptation of its functional features without a prior definition of the domain, problem, and particular task. In this case, a DIS may even be considered as a step toward artificial general intelligence. Still, in some types of DIS, centralized control (K≠∅) is presented, e.g., as a shared knowledge base or functional characteristics for the organization of G.
5. A DIS is considered as a system that (as a whole) acts as an information system with particular purposes, functional properties, performance measures, etc. Commonly, the functional characteristics are defined as a reaction of the whole DIS to the environment E based on the individual behavior of the agents (Definition 5). As a result, emergent structures within a DIS as a complex system can directly influence its performance and functional characteristics.

### 3.2. Generalized Formalism

Necessary components, providing particularities of DIS complexity, are multiple agents (both NIA and AIA), connected by multiplex and showing adaptive dynamics (see Definition 1). Each NIA has a unique counter-party AIA, which satisfies it by the suggestion of events from E and gets feedback from NIA. This local dynamic is complicated by the networking of AIA, contributing to recommendation results.

**Definition** **2.** *Agent network is a graph*G(t)*—dynamic weighted duplex, such that*G(t)=(V(t),E(t),W): $V=V_{1}\cup V_{2},E=E_{1}\cup E_{2}\cup E_{3}$*—sets of nodes and edges, where nodes correspond to NIA and AIA, and edges connect NIA with its counter-party AIA and reflect the interconnections of AIA affecting DIS properties. i.e.,*

1. $V_{1}\left(t\right)\subseteq \left\{a_{i}\left(t\right)\right\},V_{2}\left(t\right)\subseteq \left\{u_{i}\left(t\right)\right\},$
2. $\forall i\in \left\{1;2\right\}E_{i}\left(t\right)\subseteq V_{i}\left(t\right)\times V_{i}\left(t\right)$, $E_{3}=\left\{\left(u,v\right)\;|\;u\in V_{1},v\in V_{2}\right\}$
3. $W=\left\{w_{ij}^{k}\right\},\;w:E\to \mathbb{R}$*, which may change with time,*
4. $\forall u\in V_{1}\;\exists !v\in V_{2}$*—multiplex condition, i.e., *$w_{ij}^{3}=1\Rightarrow \left(\forall k\;w_{ik}^{3}=0,\;w_{kj}^{3}=0\right)$*. For simplicity, let fix *$\forall i=1..m\;w_{ii}^{3}=1.$

NIA, $\left\{u_{i}\right\}$, are characterized by their intentions (Definition 4) and interaction rules (Definition 3) with environments. Then, dynamics of interactions between NIA and events are driven by reaction and effect functions:

**Definition** **3.** *NIA feedback functions. Let* $E\subset {\mathbb{R}}^{n}$ *be an event environment,* $u\left(t\right)\in {\mathbb{R}}^{m}$ *is an agent state at discrete time stamp* t *. Then*

1. *Event* e∈E *affects agent state by an update function as* u(t+1)=f(u(t),e) *;*
2. *Agent attitude to an event is determined by the reaction function*$r\left(u\left(t\right),\;e\right):{\mathbb{R}}^{n}\times {\mathbb{R}}^{m}\to \mathbb{R}$.

Then, for continuous time, the aggregated effect of all events for the considered simulation interval [t−T, t] is:

(1) $$u\left(t\right)=\;\int_{t-T}^{t}\left[f\left(u\left(\tau \right),e\left(\tau \right)\right)-u\left(\tau \right)\right]\;d\tau .$$

**Definition** **4.** *NIA is* $\forall \;t\in \mathbb{N}\;u_{i}\left(t\right)=\langle u_{i}\left(t\right),\;u_{i}^{*},\;f,r,E\rangle$ *, where*

*(1)*  $u_{i}\left(t\right)\in {\mathbb{R}}^{m}$*—state variable,*$u_{i}^{*}\in {\mathbb{R}}^{m}$*is objective, such that*$u_{i}$*tends to reach its objective for minimal time*t*:*$\;u_{i}^{*}\left(t\right)-u_{i}\left(t\right)\underset{t\to min}{\to }min$;
*(2)*  $E\subset {\mathbb{R}}^{n}$ *is the event environment,* f *and* r *are the update and reaction feedback functions, affecting dynamics of NIA states.*

In this way, $u_{i}$ is a model of an NIA, observed via state variable and changing due to interactions with the environment (Definition 3) to achieve goals. Then, artificial intelligence agents (AIA) are their counterparts, optimizing routes to the goals by suggestions of optimal events. That is, AIAs comprise NIA representation, environment projection, and a decision-making algorithm A, providing optimal events as output.

**Definition** **5.** *Artificial intelligence agent:*$a_{i}=\langle u_{i}\left(t\right),\;u_{i}^{*},\;E,\;A\rangle$:

(2) $$\forall t\;A\left(u_{i}\left(t\right),u_{i}^{*},E\left(t\right)\right)=e_{k},e_{k}\in E:\;e_{k}={\mathrm{argmin}}_{E}\;\int_{T}\left|f\left(u_{i}\left(\tau -1\right),e_{k-1}\right)-u_{i}^{*}\right|d\tau .$$

This aims to satisfy NIA in term of its objective $u^{*}$ for the simulation interval. Since events can be recommended by sets per iteration, they are enumerated with k here. Nevertheless, furtherly, we imply one event for a timestamp and write $e_{t}$, meaning event, recommended at time t, and $e_{k}$ for a specific event chosen from E. In this way, there is a co-evolution of NIA states and AIA response, which aims at providing optimal control. Output events affect NIA states, which further affects output events.

Reinforcement contributes to AIA decision by reactions $r\left(u_{i},\;e_{k}\right)$ of NIA $u_{i}$ on output events $e_{k}$. This is usually formalized as the Markov decision process [97] in single-agent reinforcement learning and Q-learning [98]. In the DIS context, we emphasize the following dynamics, reflecting an increase of events probability to be chosen in the case of higher average reactions.

**Definition** **6.** *Reinforcement. Let* E *be an event environment,* $u_{i}$ *be an NIA with reactions* $r\left(u_{i}\left(t\right),e_{t}\right)$ *of weight* $\omega_{j}$ *; then, the preference of event* $e_{k}$ *for NIA with state* $u_{i}\left(t\right)\;$ *at the time (t + 1) depends on the previous step as*

(3) $$p\left(e_{k},u_{i}\left(t+1\right)\right)=\frac{\sum_{j=1}^{t}\omega_{j}}{\sum_{j=1}^{t+1}\omega_{j}}\;p\left(e_{k},u_{i}\left(t\right)\right)+\frac{\omega_{t+1}}{\sum_{j=1}^{t+1}\;\omega_{j}}\;r\left(u_{i}\left(t+1\right),e_{k}\right).$$

In matrix form with equal weights of reactions, it is

(4) $$P_{i}\left(t+1\right)=\frac{t}{t+1}P_{i}\left(t\right)+\frac{1}{t+1}R_{i}$$

where the reaction vector $R_{i}=\left\{r\left(u_{i},e_{k}\right)\right\}$, $P_{i}\left(t\right)=\left\{p\left(e_{t},u_{i}\left(t\right),t\right)\right\}$ is a column-vector, corresponding to $u_{i}$ preferences. Then,

(5) $$e_{t+1}={\mathrm{argmax}}_{E}\;P_{i}\left(t+1\right).$$

During decision making, an agent operates by its own information, which can be expanded by (a) their own knowledge application and (b) by information available from other agents by communication channels, which are represented by a graph.

Interactions through a weighted network of AIA $W^{1}$ contribute to reinforcement dynamics, which are formalized in Definition 6, as follows:

(6) $$P_{i}\left(t+1\right)=\frac{t}{t+1}P_{i}\left(t\right)+\frac{1}{t+1}[\alpha R_{i}+\left(1-\alpha \right)PW_{i}^{1}{}^{T}]$$

where P is a matrix of event preferences, provided by all AIA, R—reactions dependent on NIA states, and $W_{i}^{1}$—weights of AIA interaction network, related to *i*th AIA.

In this way, we consider the influence of neighbors’ and our own decisions on the further dynamics of intelligent algorithm outputs, which is complicated by the uncertainty of output choice (Equation (5)). In addition, this output is restricted by personal preference matrix P, and it is also dependent on NIA reactions $R_{i}$ and optimizations of their objectives. 

The system of Equations (5) and (6) describes DIS dynamics. NIA is analog of the external environment to the subnetwork of AIAs, but these environments are multiple and individual for each AIA, such that each AIA adapts to their NIA. The AIA state in this way can be associated with an event chosen, and the probability of its change is proportional to P from Equation (6), which is dependent on the dynamics of NIA state affected by events:

(7) $$\{\begin{array}{c} p\left(e_{k},u_{i}\left(t+1\right)\right)=\alpha \cdot \left[\frac{\sum_{j=1}^{t}\omega_{j}}{\sum_{j=1}^{t+1}\omega_{j}}\;p\left(e_{k},u_{i}\left(t\right)\right)+\frac{\omega_{t+1}}{\sum_{j=1}^{t+1}\;\omega_{j}}\;r\left(u_{i}\left(t+1\right),e_{k}\right)\right]+\\ +\left(1-\alpha \right)\cdot \underset{j\neq i}{\sum }w_{ij}^{1}\cdot p\left(e_{k},u_{j}\left(t\right)\right),\;e_{k}\in E\\ e_{t+1}={\mathrm{argmax}}_{E}\;p\left(e_{k},u_{i}\left(t\right)\right) \end{array}.$$

## 4. Case Study: A Network of Personal Self-Adaptive Educational Assistants (Avatars)

As a basis for this simulation study of the system, as described by Equations (1)–(7), we consider an example of DIS, which is implemented at ITMO University in the frames of the Avatar project (avatar.itmo.ru). It is a platform of personal self-adaptive educational assistants (avatars) interacting with each other and with its owners. This project is designed to help the students to reduce the amount of routine work during their educational activities, especially in online regimes due to pandemics of COVID-19. It allows improving the corporate and managerial culture of the university and influencing the owners of avatars on the development of the university environment by voluntarily providing data on their personal experience of participating in events and processes. Avatar is connected to a system of digital services that compete for the attention of the avatar owner in the course of providing them with content, recommendations, surveys, work results, and so on. Avatar uses the platform recommendation mechanisms to perform a high-level ranking of service outputs and their output via the feed, chat, push notifications, and other users interface elements. 

In the 2020–2021 academic year, ITMO University is implementing a digital transformation of its activities by switching to the use of role-playing professional avatars of students and employees. The ITMO Avatar project is implemented as a mobile application based on the digital identity ecosystem platform and involves launching bachelor and master students’ avatars in pilot operation from 1 September 2020. The pilot audience of the project includes 600 bachelor and master students, which are equipped with their avatars to form basic NIA–AIA pairs. For this example, NIA is a student, AIA is an avatar, and the goal of AIA is to increase the satisfaction of its NIA while learning in the environment of other AIAs and NIAs.

### 4.1. Problem Formulation

**Figure 1:**
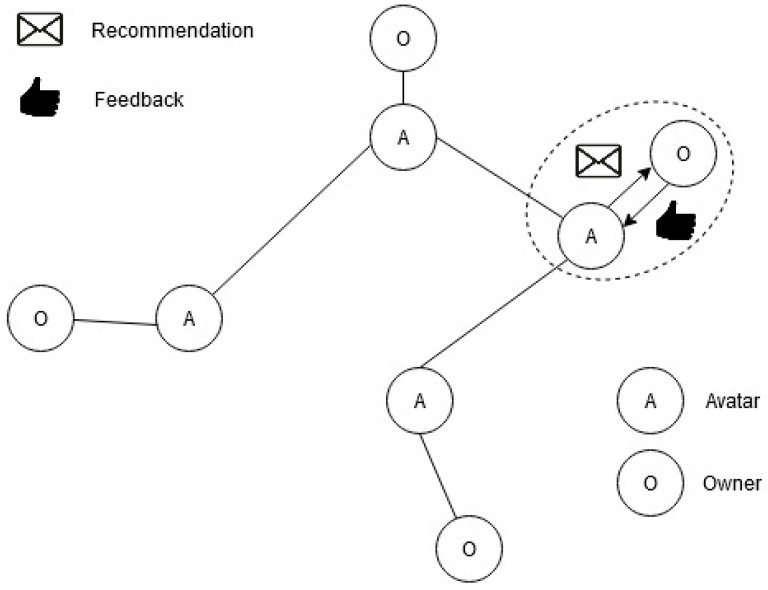
Topology of a distributed intelligent system of digital avatars (fragment).

The general structure of the DIS of digital avatars is shown in Figure 1.

The scheme implies the existence of two types of agents in DIS: NIAs (owners), $\left\{u_{i}\right\}$, and AIAs (digital assistants or “avatars”), $\left\{a_{i}\right\}$. DIS consists of a set of pairs (avatar, owner) connected by an “avatar–avatar” graph. The interaction of an avatar with its owner is described as follows: the avatar sends the recommendations $\left\{e_{k}\right\}\subset E$ to the owner, the owner evaluates the recommendations of the avatar as $r\left(u_{i}\left(t\right),e_{k}\right)$. In this way, recommendations and owners are supposed to be in the same vector space, and avatars guess their owner states by their responses. The goal of the avatar is to maximize the average amount of the owner’s ratings for recommendations (owner satisfaction). The goal of DIS is to maximize average owner satisfaction.

A highly simplified model will be considered here. However, it can illustrate the fundamental laws of such systems. Let us consider three types of information messages available to avatars—messages for “physicists” (type 1, $e_{1}=\;-1$), messages for “artists” (type 2, $e_{2}=1$), and neutral messages (type 0, $e_{0}=0$) and three corresponding types of owners. At each iteration of the simulation, the avatar chooses the type of message to show to its owner. After receiving the message from the avatar, the user can rate the message by giving it a “like”. For simplicity, we assume that the owners like the message of each type with fixed probabilities and the probability of liking the message of the matching type, $\varphi_{1},$ is several times higher than the other types of messages, $\varphi_{2}$:

(8) $$r\left(u_{i},e_{k}\right)=\;\{\begin{array}{c} 1\;with\;\varphi_{1},\;if\;u_{i}=e_{k}\\ 1\;with\;\varphi_{2},\;if\;u_{i}\neq e_{k}\\ 0,\;otherwise \end{array}.$$

Then, for fixed probabilities of liking, the expected reward per message is proportional to

(9) $$r\left(u_{i},e_{k}\right)=\;\varphi_{1}\cdot \left(1-\frac{\left|u_{i}-e_{k}\right|\cdot \left(2-\left|u_{i}\cdot e_{k}\right|\right)}{2}\right)+\;\varphi_{2}\cdot \frac{\left|u_{i}-e_{k}\right|\cdot \left(2-\left|u_{i}\cdot e_{k}\right|\right)}{2}.$$

In this way, the message type is affected by the owner’s marks $r_{i}$, and the avatar’s view of the owner state is represented by “like” sets associated with each kind of message. 

For this example, the general approach described in Section 3 is reduced to *Q*-learning. The simplest method for solving this problem is the ε-greedy algorithm. Its essence is to use both exploitation (sending messages of the type for which the largest number of likes at the moment is registered) and exploration (searching for the type that will bring more likes). In this case, Equations (3), (4), and (6) are modified, resulting in the following approach:

1. *q*-values for all types of messages are calculated:
  $$Q_{k}\left(a\right)=\frac{1}{k}\left(r_{1}+r_{2}+\ldots +r_{k}\right)$$
2. For each type of message, the average value of the *q*-value by neighbors is calculated:
  $$Q_{i}^{neighb}\left(a\right)\;=\;\underset{j\in N\left(i\right)}{\sum }Q_{j}\left(a\right)$$
3. The final value of *q*-values for the *i*-th owner is formed as a linear combination of its own Q-values and *q*-values of its neighbors:
  $${\tilde{Q}}_{i}\left(a\right)\;=\;\left(1-\alpha \right)\cdot Q_{i}^{neighb}\left(a\right)\;+\;\alpha \;\cdot Q_{i}\left(a\right)$$
  where α is the predefined mixing factor (α = 0—use only neighbor data; α = 1—do not use the neighbor’s data).
4. Then, the ε-greedy algorithm (ε = 0.1) is followed.

The dynamics, in this case, is related to updates of Q-functions for different message types, depending on AIA and NIA states:

(10) $$Q_{t+1}\left(e_{j}\right)\;=\frac{t}{t+1}Q_{t}\left(e_{j}\right)+\frac{1}{t+1}r\left(u_{i},e_{j}\right).$$

Involving the opinion of neighbors can be useful to speed up the convergence process toward the formation of the maximum value of the NIA’s satisfaction. Intuitively, the use of information from neighbors will be more useful the higher the level of assortativity in the graph (when avatars of similar owners are linked). 

In this way, Equation (7) is modified by an additional term, initiating emergence dynamics by dependence on neighboring states:

(11) $$Q_{t+1}\left(u_{i},e_{k}\right)\;=\left[\frac{t}{t+1}Q_{t}\left(e_{k}\right)+\frac{1}{t+1}r\left(u_{i},e_{k}\right)\;\right]\cdot \alpha +\frac{\left(1-\alpha \right)}{\sum_{j}w_{ij}}\sum_{j}w_{ij}Q_{t}\left(u_{i},e_{k}\right).$$

In this statement, we assume that owner satisfaction with the avatar is proportional to the number of likes. The average owner satisfaction at step *T* is calculated as:

(12) $$W\left(T\right)=\;\frac{N\left(like\right)}{T}=\frac{\sum_{E,\;T}r\left(u_{i},e_{k}\;\right)}{T}.$$

### 4.2. Computational Experiment

In this study, we address the following question: *How quickly will DIS adapt to owners ‘ preferences so that they are satisfied?* The *learning time* was introduced to answer this question. It is defined as the number of iterations of the algorithm needed to achieve an average user satisfaction value of X% of the maximum possible value.

We highlight several parameters that determine the behavior of DIS. First, we consider the question of how avatars should be linked depending on the type of their owners. For the quantitative description, the assortativity measure (class correlation between connected vertices according to [99]) was used. Second, we examine how does the number and distribution of avatar–avatar links affect the learning time. Both of them correspond to a source of complexity arising from the system scale, which implies heterogeneity of connections between cognitive agents and different network generation mechanisms. At the same time, in this motivational example, NIA has a restricted level of autonomy being predefined by the initial state.

A degree-corrected stochastic block-model [100] was chosen for generating a network of avatars because it allows varying the owners’ involvement in communities flexibly. The probability of a graph *G* in this model is described as follows:

(13) $$P\left(G|\theta ,\omega ,g\right)=\prod_{i<j}\frac{{\left(\theta_{i}\theta_{j}\omega_{g_{i}g_{j}}\right)}^{A_{ij}}}{A_{ij}!}\exp\left(-\theta_{i}\theta_{j}\omega_{g_{i}g_{j}}\right)\prod_{i}\frac{{\left(\frac{1}{2}\theta_{i}^{2}\omega_{g_{i}g_{i}}\right)}^{A_{ii}/2}}{\left(A_{ii}/2\right)!}\exp\left(-\frac{1}{2}\theta_{i}^{2}\omega_{g_{i}g_{i}}\right)\;.$$

This model includes a set of parameters *θ_i_* controlling the expected degrees of vertices *i* and parameters *ω_rs_* describing the expected value of the adjacency matrix element *A_ij_* for vertices *i* and *j* lying in groups *r* and *s*, respectively. In this work, the degrees of vertices were generated using the power function with factor ***γ*** by rejection sampling with a constraint d < d_max_, and the *ω_rs_* values were defined as follows: $w_{rr}=\;p$, $w_{rs}\left(r\neq s\right)\;=\;1-p$, so the probability of an avatar–avatar link differs only in the case where the owner types are the same.

**Figure 2:**
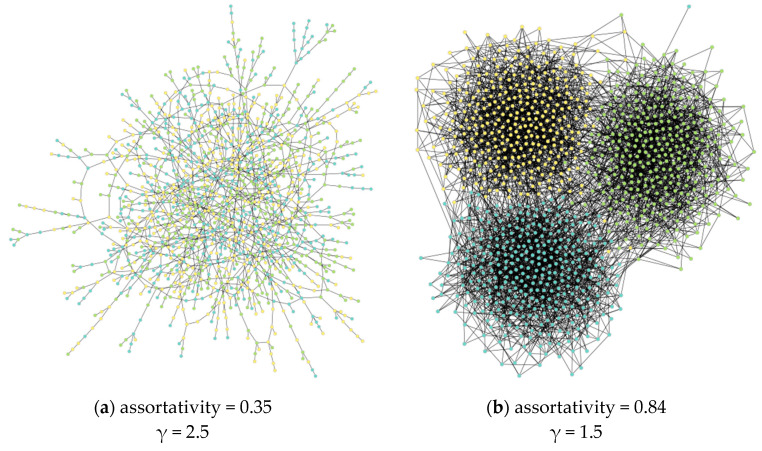
The considered networks with different block structures and mixing patterns.

**Figure 3:**
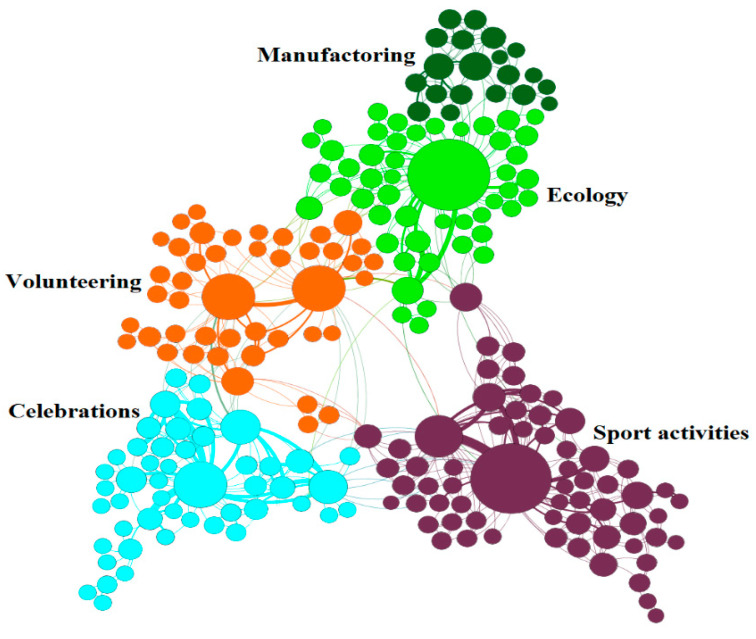
Fragment of keyword network from news portal of ITMO University.

A series of experiments were conducted to study the dependence of learning time on the ***γ*** and assortativity parameters for different mixing factors of the algorithm. For clarity, graphs of a couple of configurations are shown in Figure 2. In this simplified model, we assume that the agent is characterized by a single categorical state representing the main direction of user interests. Figure 3 represents part of the real structure of interests’ graph recovered from the news portal of ITMO University. Each news item is characterized by a set of editorial keywords, and co-occurrences of keywords form connections between them. A Loivain modularity detection algorithm is applied for a resulting graph, with the pruning of rare keywords. Figure 3 shows that, in general, a user will be represented with an attribute vector showing his/her closeness to one of the modules of the interest graph.

**Figure 4:**
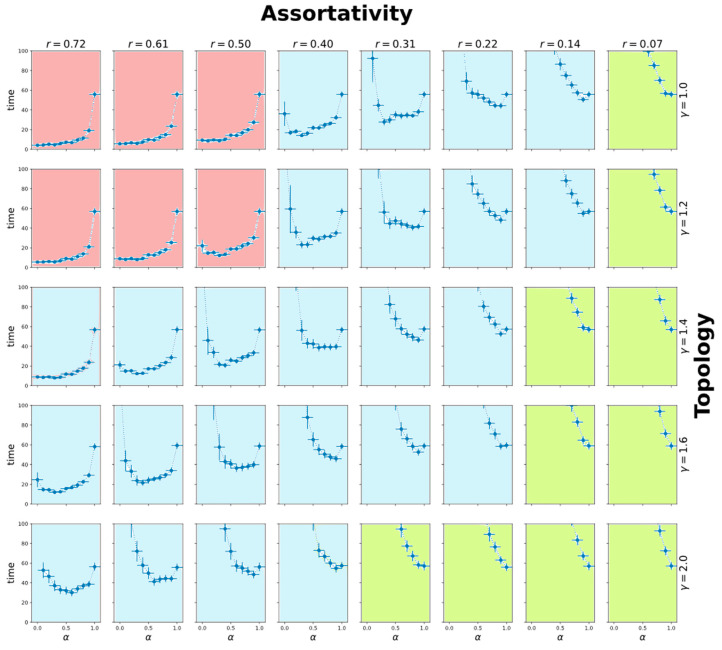
The dependences of the learning time (iterations) on the mixing factor for various γ and assortativities (red background is for Mode 1, blue background is for Mode 2, and green background is for Mode 3).

Figure 4 shows the dependences of the time needed for the system to reach 85% of the maximum owner satisfaction level for different values of the mixing coefficient, different degree distributions of vertices, and different assortativities. 

One can notice that the system has three modes:

- Mode 1—including owner knowledge worsens the speed of convergence, is observed for high assortativity values (red);
- Mode 2—limited usage of neighbors’ knowledge improves the speed of convergence (blue);
- Mode 3—including knowledge about neighbors leads to worsening the speed of convergence, is observed for low assortativity values, when the majority of neighbors give bad advices to the assistant (green).

**Figure 5:**
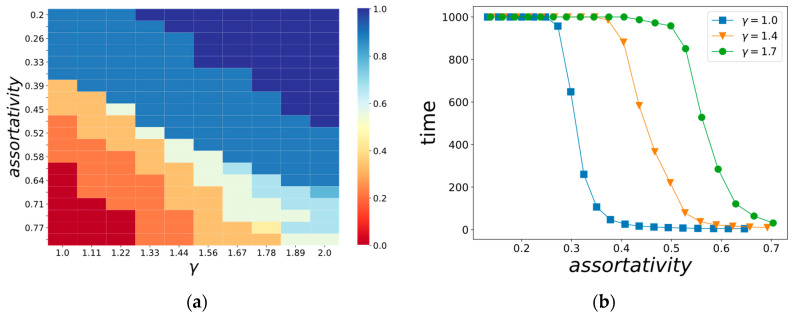
(**a**) Dependence of the optimal mixing coefficient α for various topologies and network assortativities; (**b**) learning time dependence on the assortativity for α = 0 and for several γ values.

This can even more clearly seen if we consider the dependence of the optimal mixing coefficient α on the topology and correlation measures (Figure 5a). For different DIS network topologies, the mode boundaries are shifted. This means that for a particular DIS, one cannot choose the preferred regime of learning of individual agents without considering the topology of connections for the agent.

This means that the optimal configuration of DIS cannot be found without taking into account the structure of links and the order of interaction of elements. If the topology of the AIA agents’ network is changed without reconfiguring the parameters of the AI decision-making model, the quality of recommendations will degrade. In other words, even an initially optimally configured AI will perform worse over time.

Figure 5b demonstrates another effect for the case when an agent entirely trusts its neighbors (α = 0). This figure depicts the dependency of convergence speed. The higher the assortativity, the more similar neighbors are to a given agent. Different lines correspond to different degree distributions. We may see that for each particular network topology, there is a point after which increasing the average dissimilarity of neighbors leads to a sharp slowdown of a convergence rate. This means that it may be dangerous for AIA to use all the information coming from the network without consideration of if it is useful to achieve AIA goals (e.g., to increase the satisfaction of its owner) or not. 

The effect of critical transition in Figure 5b is observed across different network sizes and parameters of nodes’ degree distribution. It shows us that even for DIS having relatively simple rules of NIA and AIA interaction and limited diversity of agents’ states, one may observe complexity effects, which may lead to non-predictable behavior of DIS. Figure 5b also shows that a topology of a network of personal self-adaptive educational assistants is connected to overall stability: the larger γ is, the larger is the threshold assortativity value, which has to be supported for fast convergence. This means that for a large γ, it is easier to violate the required assortativity level by the targeted intrusion of controlled AIA into a network of assistants, and, as a result, DIS itself is less stable.

Thus, the results above form the general control strategy of information exchange in avatar networks to avoid the unsupervised collective behavior inspired by AIA and increase the confidence of personal recommendations.

## 5. Discussion

### 5.1. Emergence of Complexity in DIS

As we have shown in Section 4, DIS has an intrinsic connection with a notion of complexity, including such effects as critical transitions and nonlinearity, limited predictability, self-organization, and evolutionary dynamics. Usually, complexity studies are performed by creating and experimenting with explanatory models (e.g., [101] and our models in Section 4) when the essence of some complexity phenomena is investigated for relatively simple models of agents and rules of their behavior. The simplicity assumption is required for a possibility to interpret observed systemic behavior. 

Our DIS explanatory study showed that motivational models of DIS demonstrate distinctive complexity effects, in particular:

(1) Limited predictability of regimes. The regimes of a system of interacting digital avatars (Modes 1–3 in Section 4) determine the optimal strategy for individual AIAs. In turn, the actual regime for a given DIS is determined by a topology of connections. This implies that AIA needs to have information about global DIS topology to find an optimal recommendation strategy. In this case, limited predictability is caused by the fact that it is hard to predict the optimal mixing coefficient before examining the quality of AIA decisions for given network topology. In this motivational model, mixing coefficients are homogeneous across the agents. However, a more realistic case is when AIAs may also tune their mixing coefficient according to the knowledge about the similarity of neighboring NIA to their owner. The interesting question arises about how to find a locally optimal mixing coefficient and which knowledge is necessary for fine-tuning of that coefficient (only nearest neighbors, several hops, or whole network). Another question may be to study heterogeneous models of satisfaction of NIA (assistant owners) because different people may be characterized by different tolerance levels to unsuitable recommendations. Thus, further studies may include the problems of optimal AIA control search as well as learning data-driven models of NIA and AIA interactions.

(2) Existence of tipping points for “maximum trust” case. For α=0, AIAs use only information from neighbors and neglect feedback from their owner. This case means that the assistant owner totally believes in its environment. We have shown that for this “maximum trust” case, there exists a critical value of dissimilarity of the agent’s environment, after which convergence starts to slow down sharply. This means that after a certain dissimilarity level, it becomes dangerous for NIA to believe its environment. This effect raises an issue of the trustworthiness of algorithms that are trained by data coming from other agents of DIS. Moreover, different AIA may be controlled by the same NIA (such as botnets). In such a case, it becomes possible to influence the learning of other AIAs by transferring false information about the quality of the content within the network. In some sense, it is another implementation of the adversarial attack performed not by submitting a specific object to the inference mechanism but by providing specific responses from the environment of the agent. 

Summarizing, we were able to show that even a relatively simple motivational model of DIS demonstrates distinctive complexity properties. This is supported not only by the results of the experiments but also by the structure of the proposed model of DIS. Emergent phenomena are related to feedback links between different components at the micro level. Table 2 demonstrates the differences in factors contributing to node states modification and adjacency matrix evolution. In this way, one can distinguish the influence of node states at previous iterations, the contribution of neighbors in combination with topological effect, and dynamics of the interaction network. These factors contribute to emergent complexity in different degrees, which is related to uncertainty and to the number of influential components. Here, $\varphi_{k}$—oscillator state, $w_{ij}$—connection weight for pairs of oscillators, ε—coefficient of edge weight modification, f,g, V—functions of own, pairwise, and edge contribution to oscillator state dynamics, S,I—discrete state variables for diffusion dynamics model, r—infection rate, p—the probability to have infected neighbor, $\left\{e_{k}\right\}$—a set of discrete state variables of DIS nodes, α—parameter shifting own and neighboring contribution.

The dynamics of a DIS is complicated by several influential factors, containing probabilistic behavior. Although network topology is static, the changeable probability of state modification contributes as its own agent state with α coefficient and as probabilities of other agents with (1−α). The dynamical probability of state change seems to introduce a new complexity level to DIS in contrast to other types of systems [105]. If, in previous cases, we observed an expected state change arising from interactions, now we see that the changes also depend on dynamics. This positively opens new promising horizons in the frame of increasing the topicality of distributed intelligent systems and emergent phenomena research.

### 5.2. Practical Implications of DIS Complexity Effects

As DIS demonstrates complexity properties, their behavior may be highly unpredictable. In turn, this behavior determines the operational characteristics of NIA–AIA networks during system evolution. These operational characteristics are quantified in the information systems field with a variety of performance measures (see, e.g., [16,17]), which may be local (agent-level), global (network-level), or external (environment-level). Table 3 shows the relation between observable quality measures, their level, and related complexity aspects that may influence the values of these measures. 

As Table 3 shows, different complexity aspects may be observed across different levels of a system and may influence quality measures for DIS users and stakeholders. This suggests that providers of DIS ecosystems (for example, this may be suppliers of digital platforms for human–AI collaboration, from expert systems to city superapps) should account for emergent effects while developing instances of algorithms to be used in tight connection with humans. When the DIS ecosystem is developed by a single supplier, this problem could be solved by creating data-driven simulators of NIA–AIA collaborative actions. These simulators use large amounts of data about registered events of NIA and AIA to train predictive models of their behavior, usually “gray” or “black boxes” (e.g., neural network classifier). In such a case, the possibility to control and to tune strategies of AIA agents to reach appropriate values of quality measures (e.g., user satisfaction) is achievable because all artificial agents belong to a single stakeholder (provider of the ecosystem itself). 

However, we assume that future problems of DIS would be related mainly to the scenarios when AIA within the DIS are supplied with different providers, and this would state the necessity for the AIA algorithm developer to find an optimal strategy of the behavior of AIA which lives in an environment with AIA from other providers. This means that AIA should not only solve the problem of increasing the satisfaction of its human owner but also the problem of creating its own representation of this environment in terms of AIA and NIA oals, strategies, data quality, and utility. In this sense, multi-provider DIS problems are relatively close to artificial general intelligence problems about searching for the balance between exploration and exploitation, particularly considered in the field of multiagent reinforcement learning. The difference here is that AIA in DIS operates not in physical locations (as robots do) and not in a 3D model of a location (as a player in a computer game does) but cyberspace when they explore and exploit flows of data about responses of other entities to the situation in a shared environment. The overall success and applicability of these networked human–algorithmic systems would largely depend on the level in which we will be able to predict and to use emergent effects from such intensive data exchange between entities with rational and irrational behavior. 

Another possibility to overcome the unpredictable behavior of the DIS is to model it at a macroscopic level by using methods and approaches of statistical and quantum physics [106]. In this case, each of the nodes may be represented as some discrete structured (spin-like) system. We can account for the interaction with the external information field in the framework of laser-like models. The temperature is a macroscopic parameter that describes various uncertainty in behavioral properties of coupled AIA + NIA network systems without extra (microscopic) specification. An assortativity that appears in the system may be recognized by means of a phase transition that leads to the formation of some social polarization (within the infinite time) and which accounts for various (macroscopic) parameters of the system [89]. In a purely quantum domain, we can assume that the temperature approaches zero. It is interesting that some properties of complex networks, operating in the anomalous regime, cannot destroy (spin) system ordering even at finite temperatures [106]. The search for conditions requiring the establishment of social (spontaneous) polarization in the presence of some non-equilibrium (time-dependent) effects represents an important but non-trivial task. We are going to represent relevant results elsewhere. 

## 6. Conclusions

Interest in IS grows increasingly. On the one hand, there are descriptions comprising heterogeneous functional components, such as knowledge bases and logical instruments. On the other hand, multiagent learning becomes more popular. Some studies use problem statements with multiple agents to increase computational efficiency, while others explore the effects of network topology on learning performance. In the case of information flows, denser networks lead to higher learning speeds and resulting quality. Nevertheless, resource and privacy restrictions result in preferences of topological design for systems of intelligent agents. In addition, reinforcement dynamics of interaction with counter-parties introduce additional complexity sources. All these studies contribute to the DIS area from different sides, bringing theoretical and empirical materials. Nevertheless, they lack general formalism, resulting in explanation and possible horizons of what is new in these topical combinations and what to expect. This we do in current research.

Different functions of artificial intelligence agents, their interactions with NIAs, and interactions with each other within a network are the primary sources of uncertainties and emerging complexity in DIS. They are formally described and connected by a dynamic systems of DIS evolution. We show that the main difference of DIS dynamics with other popular representations of complex systems is in changeable probabilities of recommendations, affected by multiple feedback links. This seems to bring a new complexity factor, additional to classic emergent phenomena (rising from states and interaction networks), resulting in qualitatively new observations.

In this work, we performed an experiment that reflects non-trivial features of the combined NIAs and AIAs (digital assistants or “avatars”) system. In the offered new model of DIS, which consists of a set of “avatar–owner” pairs connected by an “avatar–avatar” graph, the goal of DIS is to maximize the average owner satisfaction as a result of NIA–AIA interaction. We have shown that the DIS system exhibits complex behavior even for simple interaction rules established initially between the NIA and AIA. The results show that the dynamics of a DIS is complicated due to the interplay between the agents’ probabilistic behavior and network topology, which was considered as static. 

Obviously, growing and dynamic networks, related to the Internet, WWW, and other real-world networks evoke primary interest and might be the next important step in the study of the complexity problem with DIS. However, in this case, a significant increase in the complexity of NIA and AIA modeling task, which accounts their interaction, and their impact, is expected. Apparently, in this situation, it makes sense to use quantum formalism for DIS modeling, which offers an appropriate mathematical tool for account for the complexity of the system by means of interference (and, probably, entanglement) phenomena in agents’ behavior and decision making [78]. For example, the model of the interaction between the spins, placed in complex network nodes and connected by means of quantized (transverse) and classical (local) fields, contributes to the so-called super-radiant second-order phase transition, which leads to the occurrence of the spontaneous non-vanishing transverse polarization of a spin system [107]. This model can be extended as a model of opinion formation, where the AIA impact is realized by means of a quantized transverse field [89]. We are going to adapt such a model for DIS purposes in the forthcoming works.

## Figures and Tables

**Table 1:** Comparison of related systems by their focuses.

	Explicit Knowledge Base/Inference	Systemic Intelligence	Agent Intelligence	Multiple Agents	Networked	Systemic Complexity	AIA + NIA
Sensor networks	- ^1^	-	-	+	+	-	-
Ensemble learning	-	-	+	+/−	-	-	-
Complex networks	-	-	-	+	+	+	-
Quantum channels and networks	-	-	-	+/−	+	-	-
MAS	-	+/−	-	+	-	+/−	-
Distributed expert systems	+/−	+/−	+	+	+	+	-
MARL	+/−	+	+	+	+/−	-	+

^1^ +/−: partly presented, -: not presented, +: presented.

**Table 2:** Dynamics comparison for different types of complex systems and contributing components.

System Type	Node State	Own Contribution	Neighbors Contribution to Node State Increment	Edge Weights Increment	Discrete Node State	Probability of State Change
Kuramoto oscillators [102]	$\varphi_{i}$	*const*	$w_{ij}\cdot \sin\left(\varphi_{j}-\varphi_{i}\right)$	$\varepsilon \cdot \left(\varphi_{i}-\varphi_{j}\right)$	-	-
Oscillators with coupling dynamics [103]	$\varphi_{i}$	$f\left(\varphi_{i}\right)$	$w_{ij}\cdot g\left(\varphi_{i},\varphi_{j}\right)$	$-\int \left[\ddot{w_{ij}}+\frac{dV\left(w_{ij}\right)}{w_{ij}}\right]$	-	-
SI diffusion [104]	{S, I}	-	$w_{ij}\cdot r\cdot p\left(s_{j}=I\right)$	-	Yes	Fixed, r
DIS	$\left\{e_{k}\right\}$	$\alpha \cdot r\left(u_{i}\left(t\right),e_{k}\right)$	$\left(1-\alpha \right)\cdot w_{ij}\cdot p\left(u_{j}\left(t\right),e_{k}\right)$	-	Yes	$p_{t+1}\left(u_{i},e_{k}\right)$

**Table 3:** Relation between IS quality measures and complexity aspects.

Quality Measures	Agent	Network	External	Complexity Aspects
Service quality	+^2^			Collaboration
System quality		+		Resistance to perturbations Resistance to the false response Minimal number of agents
Information quality		+	+	Information percolation/degradation
Information use	+			
User satisfaction	+			Individual strategies Collision avoidance
Individual impact	+			Control driving nodes
Workgroup impact		+		Emergent phenomena
Organizational impact		+	+	Resource management

^2^ +: presented.
